# Safety Profile of Methotrexate Therapy in Patients With Rheumatoid Arthritis

**DOI:** 10.7759/cureus.27047

**Published:** 2022-07-20

**Authors:** Syed Hassan Mustafa, Tausif Ahmad, Malab Balouch, Farah Iqbal, Talha Durrani

**Affiliations:** 1 Rheumatology, Lady Reading Hospital, Peshawar, PAK; 2 Internal Medicine, NHS Highland, Inverness, GBR; 3 Neurorehabilitation Unit, Charing Cross Hospital, London, GBR; 4 Health Department, District Headquarters Hospital, Abbottabad, PAK; 5 Department of Medicine, Khyber Teaching Hospital, Peshawar, PAK

**Keywords:** mucocutaneous, cytopenias, rheumatoid arthritis, methotrexate, side effects

## Abstract

Objective

The objective of this study was to determine the safety profile of methotrexate (MTX) therapy in patients with rheumatoid arthritis

Study design

This was a cross-sectional observational study.

Place and duration of the study

The study took place in the Division of Rheumatology, Lady Reading Hospital Peshawar, from May 2020 to August 2021.

Methodology

A total of 411 patients with rheumatoid arthritis and receiving MTX in the dose of 10-20 mg/week for at least four months were included by consecutive sampling. All patients were followed for four months for the development of cytopenias, deranged liver function tests, renal function tests, fever, and gastrointestinal upsets. Data were recorded on a pro forma.

Results

There were 237 (57.6%) females and 174 (42.4%) males. The female to male ratio was 1.4: 1. The average age of patients was 43.01 years + 17.1 SD with a range of 18-72 years. Gastrointestinal side effects were the most common, found in 49 patients (11.9%), followed by mucocutaneous side effects in 35 patients (8.5%) and fever (34 patients, 8.3%).

Conclusion

Every one in three patients developed some adverse effect within six months of methotrexate therapy. Moreover, we conclude that gastrointestinal side effects were the most common side effects seen.

## Introduction

Rheumatoid arthritis (RA) is a chronic multisystem autoimmune disease with a prevalence of 0.5-1% in the general population [1-2]. The disease primarily affects the small joints of the hands, leading to joint damage, and is thus associated with high morbidity. However, the long-term prognosis of the patients has improved significantly over the past few decades primarily due to the timely diagnosis and development of disease-modifying anti-inflammatory drugs (DMARDs) [3].

Methotrexate (MTX) is the most preferred DMARD used in the management of RA since a large proportion of patients (23%-40%) improve significantly with methotrexate monotherapy due to its low cost, proven efficacy, and years of clinical experience with its use [4]. Despite the development of biological therapies and targeted synthetic therapy, MTx is still used as the first-line therapy [5]. Moreover, it is also recommended to be used along with biologics since it reduces the probability of developing autoantibodies against these new drugs [4].

As an anti-metabolic drug, methotrexate therapy is not without side effects. Though these adverse effects are less common in RA patients as compared to oncology patients due to lower doses of methotrexate required in RA [6], some of the most common adverse effects include liver damage, myelosuppression, gastrointestinal upsets, mucocutaneous problems, and lung fibrosis. It is due to these adverse effects that many patients discontinue their methotrexate therapy [7].

The exact pathophysiology of methotrexate-induced side effects is not known. However, the development of cytopenia, mucositis, and gastrointestinal upsets demonstrate that these adverse effects develop due to a deficiency of folic acids. However, not all side effects are due to folate deficiency. For instance, the development of lung fibrosis, liver damage, and renal impairment occurs without folate deficiency. This indicates that other mechanisms may also be involved [8].

In Pakistan, methotrexate is injudiciously prescribed to patients without following the guidelines for monitoring the development of side effects of MTX therapy. Therefore, the purpose of this study is to determine the frequency of different adverse effects of methotrexate therapy in patients with rheumatoid arthritis.

## Materials and methods

This descriptive study was conducted in the Division of Rheumatology, Lady Reading Hospital Peshawar, from May 2020 to August 2021. The Rheumatology Division of Lady Reading Hospital is the only rheumatology unit in the whole province of Khyber Pakhtunkhwa. A total of 411 RA patients aged 18-72 years of either gender were included in the study through consecutive sampling techniques. RA was defined based on the American College of Radiology (ACR) 2010 criteria according to which all patients having a score of 6 or more will be classified as having RA. The criteria are given in Table 1.

**Table 1:** American College of Rheumatology (ACR) 2010 criteria Source: [9] RF: rheumatoid factor; ACPA: anti-citrullinated protein antibodies; CRP: C-reactive protein; ESR: erythrocyte sedimentation rate

Domain	Category	Point score
A	Joint involvement (0–5 points)	
	1 large joint	0
	2–10 large joints	1
	1–3 small joints (large joints not counted)	2
	4–10 small joints (large joints not counted)	3
	>10 joints including at least one small joint	5
B	Serology (at least one test needed for classification; 0–3 points)	
	Negative RF and negative ACPA	0
	Low positive RF or low positive ACPA	2
	High positive RF or high positive ACPA	3
C	Acute-phase reactants (at least one test needed for classification; 0–1 point)	
	Normal CRP and normal ESR	0
	Abnormal CRP or abnormal ESR	1
D	Duration of symptoms	
	<6 weeks	0
	≥6 weeks	1
Domain	Category	Point score
A	Joint involvement (0–5 points)	
	1 large joint	0
	2–10 large joints	1
	1–3 small joints (large joints not counted)	2
	4–10 small joints (large joints not counted)	3
	>10 joints including at least one small joint	5
B	Serology (at least one test needed for classification; 0–3 points)	
	Negative RF and negative ACPA	0
	Low positive RF or low positive ACPA	2
	High positive RF or high positive ACPA	3
C	Acute-phase reactants (at least one test needed for classification; 0–1 point)	
	Normal CRP and normal ESR	0
	Abnormal CRP or abnormal ESR	1
D	Duration of symptoms	
	<6 weeks	0
	≥6 weeks	1

The inclusion criteria were all adult patients > 18 years of age, either gender, diagnosed with rheumatoid arthritis, normal baseline liver function tests, complete blood picture, renal function tests, pulmonary function tests, and serum electrolytes.

The exclusion criteria were patients with viral hepatitis, patients taking other hepatotoxic drugs, diabetic patients, obese patients with a BMI of more than 30, patients having any other liver disease, such as hemochromatosis, Wilsons disease, and autoimmune hepatitis, patients with hematological conditions, such as leukemias, aplastic anemias, thrombocytopenias, or any history of recent blood loss, and patients having any previous obstructive or restrictive lung disease.

Approval was taken from the hospital ethical committee, informed consent was taken from all RA patients and they were subjected to detailed history and complete physical examination. Thereafter, 10 ml of blood was taken from all patients and sent to the hospital lab for liver function tests (LFTs), renal function tests (RFTs), and complete blood count (CBC). After that, all patients were subjected to spirometry, and PFTs were noted. All patients were started on methotrexate 10 mg/week and folic acid 5 mg/week, making sure that no patient takes both drugs on the same day. All patients were also given tapering steroids as bridging therapy and paracetamol/tramadol for pain relief only.

All patients were followed for four months, and the dose of methotrexate was increased up to 20 mg/week depending upon the patient’s disease status. At the end of four months, all patients were again examined for the development of any complications, plus 10 cc of blood was taken and sent to the hospital lab for CBC, RFTs, and LFTs. All patients were also subjected to spirometry again at the end of four months for pulmonary function tests (PFTs). The following abnormalities were noted as significant: alanine transaminase twice the upper limit of normal is considered hepatotoxic, serum creatinine of more than 1.29 mg/dl is considered nephrotoxicity, hemoglobin less than 12 gm/dl for males and 11.5 gm/dl for females is considered anemia, white cell count of less than 4000/ul is considered leucopenia, platelets of less than 150 x 109 /ul are considered thrombocytopenia, first second of forced expiration (FEV1)/forced vital capacity (FVC) of less than 70% is considered a pulmonary side effect, presence of or self-reported history of oral ulcers and hair loss considered mucocutaneous involvement, presence of or self-reported history of nausea, vomiting, and diarrhea considered gastrointestinal involvement, and development of or any history of any fever.

All investigations were done in the same lab and by the same doctor blinded for the study. Similarly, all the history and examination were done by a consultant physician blinded for the study. All data were recorded on a pro forma.

Data were then analyzed by SPSS 16 (SPSS Inc., Chicago, Ill). All the categorical data was calculated as frequencies and percentages. All the numerical data were calculated as mean and standard deviation. Data were stratified by age and gender. To know the significant difference, the chi-square test was used.

## Results

A total of 411 patients with rheumatoid arthritis were included in the study. There were 237 (57.6%) females and 174 (42.4%) males. The female to male ratio was 1.4: 1. The average age of patients was 43.01 years + 17.1 SD with a range of 18-72 years. Average methotrexate dose was 13.6 mg/week. Overall, 132 patients (32%) developed some side effects out of which 83 patients (35%) were females while 49 were male patients (28%). See Table 2 for the demographics of the study population. 

**Table 2:** Basic demographics of the study population

Variables	Frequency
Total patients	411 (100%)
Females	237 (57.6%)
Males	174 (42.4%)
Mean age + SD	43.01 years + 17.1
Youngest Patient	18 years
Oldest Patient	72 years
Average MTX dose	13.6 mg/week
Total patients with side effects	132 (32%)
Female patients with	83 (35%)
Male patients	49 (28%)
The average duration of treatment (in years)	2.49
Patients who stopped treatment	26 (6.3 %)

Gastrointestinal side effects were the most common, found in 49 patients (11.9%). Mucocutaneous side effects were the most common, found in 35 patients (8.5%), followed by fever (34 patients, 8.3%). All other side effects are given in Table 3.

**Table 3:** Different side effects of methotrexate

S.NO	Methotrexate side effects	Frequency
1.	Gastrointestinal	49 (11.9%)
2.	Mucocutaneous	35 (8.5%)
3.	Fever	34 (8.3%)
4.	Anemia	29 (7.05%)
5.	Hepatotoxicity	14 (3.4%)
6.	Thrombocytopenia	7 (1.7%)
7.	Leucopenia	6 (1.45%)
8.	Pancytopenia	5 (1.21%)
9.	Others	11 (2.6%)

As regards gender, 34 female patients were found to develop gastrointestinal side effects compared to 15 male patients, the difference being statistically significant (p=0.04), while mucocutaneous side effects and fever were found in 17 and 16 female patients and each in 18 male patients, respectively (p=0.28). The rest of the adverse effects are shown in Table 4.

**Table 4:** Stratification of methotrexate side effects according to gender

Methotrexate side effects	Male	Female	Total	p-value
Gastrointestinal	15 (30.6%)	34 (69.4%)	49	0.04*
Mucocutaneous	18 (51.4%)	17 (48.6%)	35	0.28
Fever	18 (53%)	16 (47%)	34	0.2
Anemia	14 (48.2%)	15 (51.8%)	29	0.56
Hepatotoxicity	5 (35.7%)	9 (64.2%)	14	0.78
Thrombocytopenia	3 (42.8%)	4 (57.2%)	7	1
Leucopenia	2 (33.3%)	4 (66.7%)	6	1
Others	4 (36.4%)	7 (63.6%)	11	0.76

A total of 26 patients stopped treatment, out of which 17 patients had developed methotrexate adverse effects while nine patients had not developed side effects yet stopped the treatment as shown in Table 5.

**Table 5:** Chi-square test: adverse effects and treatment cessation

		Adverse effects		Total	p-value
		Yes	No		
Patients who stopped treatment	Yes	17 (4.13)	9 (2.18%)	26 (6.32%)	0.001
	No	115 (27.98%)	270 (65.69%)	385 (93.67%)	
Total		132 (32.11%)	279 (67.88)	411 (100%)	

## Discussion

In this study, we observed that 132 (32%) of patients developed side effects from methotrexate therapy. Moreover, we observed that adverse effects of methotrexate were more common in females as compared to male patients, but the result was not statistically significant (p=0.08).

Solomon et al., in their study, followed 4,786 patients for 23 months [6]. Patients were randomized into two groups with one group on low-dose methotrexate, i.e. ranging from 10 mg - 20 mg/ week while the other group was on placebo. They found that 87% of the patients in the methotrexate arm developed adverse effects compared to 81% in the placebo arm, with a relative rate of 17%. In our study, 32% of the patients developed adverse effects. The vast difference between these two diseases is because they followed the patients for 23 months compared to our study, as we studied them for six months only. The cumulative incidence curve of Solomon et al. further acknowledges that the frequency of side effects increases with the duration of medicine. Our findings were similar to Bologna C et al., as they observed that 23% of patients stopped methotrexate treatment due to toxicity [10].

In this study, we observed that gastrointestinal upsets were most common with methotrexate therapy, accounting for 11.9% of patients. Asai S et al. also studied the effect of low vs high-dose methotrexate therapy on the GI system of patients and observed that 32% of patients on high dose developed reflux compared to 24% on low-dose therapy [11]. Moreover, we observed that gastrointestinal adverse effects were more common in females as compared to males. One reason for these differences is that they used the Gastrointestinal Symptom Rating Scale (GSRS) while we relied on self-reporting. In their study, Atsumi et al. reported that GI adverse effects occurred in 24% of patients on methotrexate therapy [12]. The GI adverse effects that they reported were nausea, vomiting, and reduced appetite, which were the same symptoms we looked for. However, since they followed the patients for 52 weeks, they reported a higher frequency. Solomon et al., on the other hand, reported 11.4% of GI-related adverse effects, which were similar to our findings [6]. Nanke Y et al. observed that bacterial infection of the gastrointestinal tract can occur in patients on methotrexate, which can be life-threatening [13].

The mucocutaneous adverse effects of methotrexate were found in 8.5% of the patients in our study and their occurrence was irrespective of gender. Atsumi et al. reported a 16.6% frequency of patients who developed stomatitis [12]. In another study, 13.5% were found to develop mucocutaneous adverse effects [14]. Carpenter EH et al. reported that 2% of the patients stopped their methotrexate treatment due to mucositis [15]. In our study, mucositis was never severe enough to lead to treatment discontinuation. In agreement with our study, Hoekstra et al. also observed that the mucocutaneous adverse effects of methotrexate therapy do not correlate with gender [16].

Owing to the immunosuppressive activity of methotrexate, infections have always been a concern with MTX treatment. We observed that 8.3% of patients developed a fever while on methotrexate therapy. In our study, pancytopenia was reported in five patients while six patients developed leukopenia. This is in accordance with several studies that reported an increased risk of infections in rheumatoid arthritis patients on methotrexate [17-21]. In the literature, neutropenia and pancytopenia have been reported to occur in 1.4% to 7% and 0.3% to 2.1% of the patients, respectively, which is also in accordance with our study [22-26].

We reported that hepatotoxicity following methotrexate therapy in RA patients occurred in 3.4% of patients. This is in contrast to another study, which reported a 13% elevation in alanine transaminase twice the upper level of normal in patients with methotrexate therapy [27]. They also observed that the frequency of hepatotoxicity is affected by the length of therapy, with more patients developing deranged liver functions following more than one year of therapy. Moreover, additional therapies were used, which led to a higher frequency of liver damage compared to our study. In yet another study, the incidence of developing transaminases above two times the upper limit of normal was found in only 1% of patients, which is similar to our study [28].

Methotrexate-related side effects can be minimized by the concomitant use of oral folic acid [29]. In this study, patients were on oral folic acid 5 mg/week, and the dose was given 24 hrs after the methotrexate dose. Moreover, studies have also supported the use of 5 - 10 mg of folic acid per week to be used with methotrexate therapy since it reduces methotrexate toxicity [30].

So far, little data is available regarding the frequency of different adverse effects of methotrexate in the Pakistani population. Moreover, the drug is often injudiciously prescribed by physicians without following any international guidelines. In Pakistan, this problem could partially be attributed to the handling of rheumatology patients by general physicians, in contrast to specialized rheumatologists, which are lacking in the country. Another contributing factor could be the physician's intent to get a strong and quick therapeutic response. However, neither of these causes justify the excessive use of the drug in the population. Furthermore, contrary to some of the published literature showing that the adverse effects of the drug are mild and manageable, our study showed that many patients were bothered enough by these problems that they had to switch treatments. Hence, our findings, therefore, guide the health care officials and caregivers regarding what side effects to look for in patients on methotrexate therapy.

This study has certain limitations, which include the relatively small sample size, being a unicenter study, having a short follow-up period, and a lack of randomization.

## Conclusions

We conclude that every one in three patients develops some adverse effect within six months of methotrexate therapy. However, most of them are not life-threatening, and gastrointestinal side effects were the most common side effects seen. It is worth mentioning that despite methotrexate being a preferred and cost-effective option for treating rheumatoid arthritis, every patient on methotrexate therapy should be monitored for the development of side effects during every follow-up visit.
